# Innovative Exercise in Routine Cancer Care: Insights from Eight Years of Integrated Oncological Exercise Therapy (OTT)

**DOI:** 10.1186/s40798-026-00988-0

**Published:** 2026-03-04

**Authors:** Timo Sonntag, Ariana Safi, Vera Coutellier, Anna Lorenz, Philipp Zimmer, Eva M. Zopf, Fiona Streckmann, Lars Gerland, Petra Wirtz-Derksen, Anja Großek, Anne Kollikowski, Constanze Handmann, Stefanie Siebert, Paul J. Bröckelmann, Christian P. Pallasch, Wilhelm Bloch, Thomas Elter, Michael Hallek, Damir Zubac, Freerk T. Baumann

**Affiliations:** 1 Faculty of Medicine and University Hospital Cologne, Department I of Internal Medicine, Center for Integrated Oncology Aachen Bonn Cologne Duesseldorf, University of Cologne, Cologne, Germany; 2 Division of Performance and Health, Institute for Sport and Sport Science, Technical University Dortmund, Dortmund, Germany; 3 Mary MacKillop Institute for Health Research, Australian Catholic University, Melbourne, VIC Australia; 4 Department of Medical Oncology, Cabrini Cancer Institute, Cabrini Health, Melbourne, VIC Australia; 5 Department of Sport, Exercise and Health, University of Basel, Basel, Switzerland; 6 Oncology, University Hospital Basel, Basel, Switzerland; 7 Department of Molecular and Cellular Sports Medicine, Institute of Cardiovascular Research and Sports Medicine, German Sport University Cologne, Cologne, Germany; 8 Comprehensive Cancer Center Mainfranken, University Hospital of Würzburg, Würzburg, Germany

**Keywords:** Neoplasms, Clinical care, Exercise program, Exercise oncology, Cancer patients, Physical activity

## Abstract

**Background:**

The beneficial effects of exercise in cancer patients are increasingly understood, whereas the inclusion of structured oncological exercise as a standard of care remains a challenge. Herein, we evaluate the innovative, supervised Oncological Exercise Therapy (OTT) integrated into the standard of clinical care and report patient characteristics, exercise participation and attendance, and effects on patient-reported outcomes (PROs) and physical performance.

**Methods:**

An observational study was conducted to analyze patient and exercise cohort data collected between 2012 and 2020 on the OTT. Cancer patients were encouraged to attend the personalized OTT intervention for a minimum of three months. Demographic, medical and treatment-related patient data were documented at enrollment. Exercise attendance was measured up to one year after enrollment, and exercise efficacy was evaluated between 6 and 24 weeks of exercise and included strength and endurance assessments and PROs on quality of life, fatigue, and psychosocial distress.

**Results:**

Most of the *n* = 1660 enrolled patients (median age: 54 years [18–86]) were female (70%), diagnosed with breast cancer (40%), without metastasis (80%) and were receiving anticancer treatment (65%). One-third (32%) exercised for an average of 19 ± 10 sessions in a 19-week (± 13 weeks) period. Only 1% of patients reached the recommended average of ≥ 2 weekly sessions on the OTT. Older age and shorter travel distance were associated with increased exercise attendance. Exercise improved strength and endurance performance and PROs, indicating more pronounced effects in patients with greater exercise attendance.

**Conclusions:**

Innovative exercise programs can be established as standard of cancer care in hospital settings. These real-world data suggest a beneficial effect of exercise in cancer patients on PROs and physical outcomes, with more pronounced effects in patients with greater exercise attendance. Therefore, strategies to increase exercise attendance appear crucial to maximize benefits derived from real-world exercise interventions in cancer patients.

**Supplementary Information:**

The online version contains supplementary material available at 10.1186/s40798-026-00988-0.

## Key Points

Innovative exercise programs are feasible in clinical cancer care.

Personalized exercise can benefit patients’ physical condition and patient-reported outcomes.

Real-world exercise habits do not meet exercise recommendations.

Exercise adherence seem vital to maximize the beneficial effects.

**Supplementary Information:**

The online version contains supplementary material available at 10.1186/s40798-026-00988-0.

## Background

Currently, there is large and still growing evidence supporting the role of exercise programs in the management of cancer patients both during both treatment and survivorship [1–2]. Moreover, the recently published study by Courneya et al. illustrated an improved disease-free and overall survival of colon cancer patients after adjuvant chemotherapy following a 3-year structured exercise program [3].

In contrast, the availability of established exercise programs for cancer patients is poorly developed and studied. In 2018, the American College of Sport Medicine (ACSM) roundtable identified ~ 150 existing concepts of exercise programs for cancer patients worldwide [2]. The incidence of cancer is predicted to increase globally, and 27.5 million new cases are predicted by 2040 [4]. The current knowledge-to-practice gap regarding structured exercise programs constitutes a large urgent unmet need for the translation of exercise science into exercise programs in the standard of cancer care. Well-established exercise concepts such as the *LIVESTRONG at the YMCA* program [5] or the *ActivOnco* program [6] focus mainly on cancer survivors. However, to date, there is still a lack of specifically tailored and supervised exercise programs delivered by health care professionals to patients during or shortly after acute anticancer treatment. To address this research-to-practice gap, we herein describe and analyze a unique and innovative exercise concept termed Oncological Exercise Therapy (OTT) which is integrated into the standard of care at the University Hospital Cologne, Germany. Within the framework of this supportive exercise concept, cancer patients were offered a supervised, evidence-based exercise intervention situated in a hospital setting. In the this article, real-world data gathered on the OTT between 2012 and 2020 will be reported and analyzed in an observational study design. First, we report the characteristics of patients participating in the OTT program. Second, patients’ exercise participation and attendance patterns were benchmarked with current exercise guidelines, and attendance predictors were identified. Finally, we examined the effects of OTT on physical performance measures and patient-reported outcomes (PROs), such as quality of life and fatigue.

## Methods

This observational study reports on the OTT cohort established at the University Hospital Cologne, Germany. After the development of the OTT concept, it was established in 2012 and approved by the Institutional Review Board of the Medical Faculty of the University of Cologne (No. 13–050). The study was carried out in accordance with the Code of Ethics of the World Medical Association (Declaration of Helsinki). Since 2012, the OTT has continued to recruit eligible cancer patients to the present day. For this analysis, we considered the data collected from April 2012 to February 2020 due to structural changes in the OTT facility and the implementation of COVID-19 measures from March 2020 onward.

### Observational Design

**Fig. 1:**
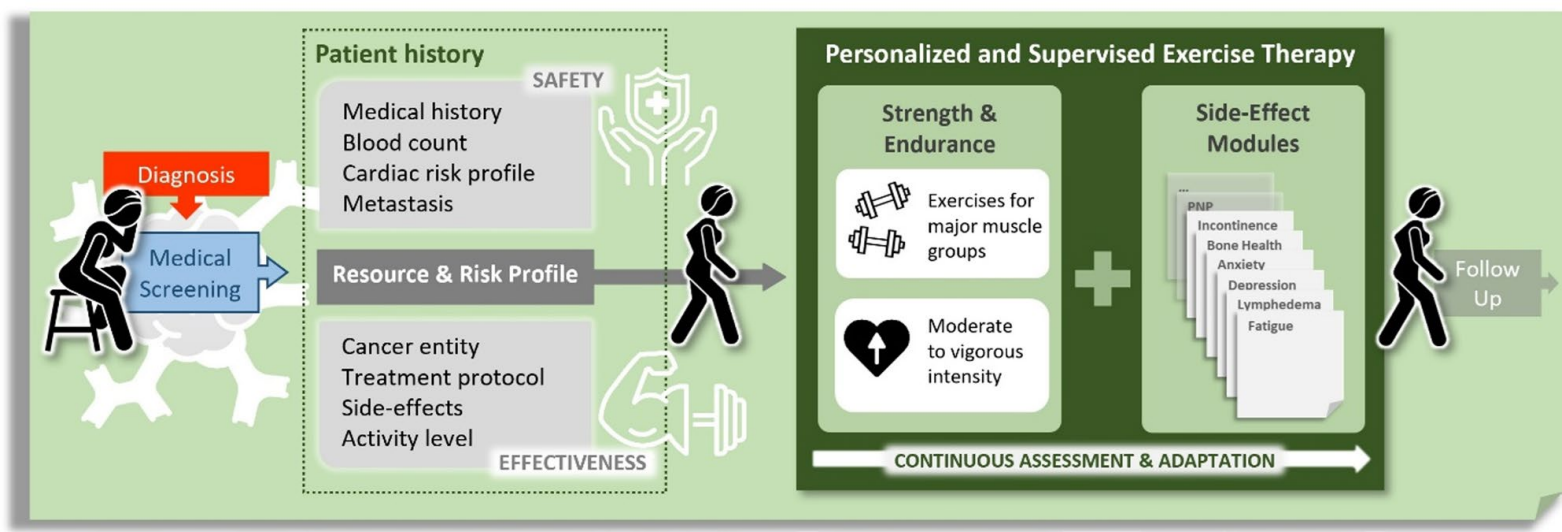
Oncological Exercise Therapy (OTT) concept

The OTT exercise concept aims to integrate evidence-based exercise interventions into a clinical exercise facility with ongoing scientific support and easy-to-access participation. The exercise concept and design were published previously [7]. The program was tailored to cancer outpatients with a medical referral and/or recommendation to an exercise program, which was given by care professionals (i.e., physicians, nurses) to patients who received any ongoing anticancer treatment (e.g., chemo-/immunotherapy, radiotherapy, surgery, stem-cell transplantation). Additionally, cancer survivors with persisting cancer-related impairments could be enrolled. Patients had to be at least 18 years old, provide written informed consent, have medical exercise clearance by a physician and be capable of visiting the OTT facility (Fig. 1).

Eligible patients were offered an individualized exercise intervention in the form of the OTT. The duration of the prescribed exercise intervention was at least 12 weeks but could be extended in cases of the aforementioned ongoing anticancer treatment or prevalent cancer-related impairments in cancer survivorship. Participation was not restricted to cancer patients treated at the University Hospital Cologne, and the program was advertised in and around Cologne, Germany, local cancer support-groups, social media channels and the homepage of the University Hospital Cologne. The exercise facility had weekday opening hours from 9 am to 2 pm. At least one exercise therapist with an oncological education supervised the exercise facility at any time.

### Patient Involvement

A patient committee, including patient advocates, was established in 2014 and has since been involved in the continued development of OTT. The patient committee was included in decisions regarding assessment, recruitment strategies and exercise methods.

### Exercise Intervention

The exercise facility of the OTT was located in a 74 m² room. The facility was equipped with a chipcard-driven Milon training circle (Milon GmbH, Germany) containing exercise devices for the major muscle groups. The general exercise program consisted of both strength (Table S1) and endurance (Table S2) training. The exercise intensities were adapted individually but generally consisted of prolonged aerobic training or anaerobic interval training for endurance training and muscle endurance or hypertrophy training for strength training (Suppl. Methods). Depending on the patients’ individual situation, specific exercise modules were added to target cancer-related impairments, such as fatigue, cachexia, incontinence or chemotherapy-induced peripheral neuropathy (CIPN; Table S3). For example, the CIPN module contained exercises on the vibration plate or balance exercises. Figure 1 illustrates the general workflow within the OTT concept.

All patients were supervised in a 1-to-1 exercise setting within the first two weeks to ensure high-quality exercise execution and avoid the risk of injuries. With progression in the exercise routine, the supervision level was reduced to exercise monitoring and intensified supervision at assessment appointments. Patients were assessed for their aerobic and strength performance and standardized PRO measurements after two weeks of familiarization and after 15-week cycles (Suppl. Methods). Patients were encouraged to exercise as per the OTT for 2–3x/week for a 60-minute exercise session each, referring to current [2] or previous [8] recommendations of the ACSM exercise guidelines.

### Data Collection and Analysis

At enrollment, baseline demographics, cancer-related information, comorbidities (Charlson Comorbidity Index, CCI [9]) and ongoing or received anticancer treatment were documented (Fig. 1). Patients were asked if they experienced any cancer-related impairments. Additionally, patients were requested to rate the subjectively perceived rate of fatigue on a visual analog scale (VAS) from 1 to 10.

Fatigue was considered present with VAS scores < 3 according to the fatigue classification for exercise prescription used by McNeely et al. [10]. This outcome assessment was established in 2014 and used onwards, indicating that Fatigue outcomes were only available in a subsample with a lower sample size.

Psychological distress, measured by the Hospital Anxiety and Depression Scale (HADS)-questionnaire [score range: 0–42], was assumed at a cutoff value of > 13 points [11]. With respect to exercise attendance, patients with more than one completed assessment were analyzed. In this study, the exercise attendance analysis was limited to the first four assessments (T0-T3). The efficacy analysis of exercise was limited to the first two assessments (T0-T1) and only to assessments with a duration of 6–24 weeks between both appointments. Additionally, patients were separated into two adherence subgroups: patients who showed an average OTT-exercise adherence of lower than one session per week (EX < 1/week) and those with one or more exercise sessions per week (EX ≥ 1/week). Assessments included strength test (hypothetic one-repetition maximum, knee curl, extension, row and bench press), endurance tests (cardiopulmonary exercise testing) and PROs on quality of life (European Organisation for Research and Treatment of Cancer [EORTC]-quality of life questionnaire [QLQ]=C30), fatigue (Multidimensional Fatigue Inventory [MFI] 20), and anxiety and depression burden (HADS) (Supplementary Material Methods).

To ensure the robustness of our dataset, we excluded patients with > 25% missing data in the history. The data are presented as the means, medians, standard deviations and interquartile ranges (IQRs), as indicated. For subgroup difference analyses, ANOVA with Tukey’s test, the Games-Howell post hoc test or the chi-square test were applied. Repeated-measures ANOVA was used to calculate exercise efficacy. Bivariate correlations were conducted to correlate attendance data with baseline demographic data.

### Equity, Diversity and Inclusion Statement

Our cohort reflects demographic and socioeconomic diversity in patients with wide inclusion criteria and a free-of-charge exercise intervention. Broad geographical diversity could not be assured due to the location of the exercise facility in Cologne.

## Results

In the period from 2012 to 2020, a total of *n* = 1788 patients were enrolled in the OTT, of whom *n* = 1660 with sufficiently complete datasets were analyzed.

### Baseline Demographic Data

The majority of the analyzed population was female (70%), with a median age of 54 years (range: 18–86), and was diagnosed with breast cancer (40%) without metastasis (80%) and undergoing acute cancer treatment (80%), with chemotherapy being the most prevalent ongoing anticancer treatment (44%) (Table 1, Suppl. Table 1). If patients had metastases (20%), bone (8.1%) and liver (7.8%) were affected most prevalently, and 8% of the patients had multiple metastatic sites. Approximately 12% of the patients had CCI-relevant comorbidities, with pulmonary diseases and concurrent secondary cancer being the most prevalent (Table 1). The most prevalent reported cancer-related impairments were fatigue (69.7%), cancer-related cognitive impairments (CRCI, 41.1%), psychosocial distress (40.0%), chronic pain (~ 31.0%) and body weight loss (~ 21%) (Fig. S1). More than 65% of the patients lived < 10 km away from the OTT facility, with a median distance of 6.9 km (range: 0–494 km) (Table S2). The median time between cancer diagnosis and OTT enrollment was 6 months (range: 0–492 months; Table S2).

**Table 1:** Participant characteristics

*N* (1660, total study population)	No.
*Sex (data available, 1656, 99.7%)*	
Men	490 (29.5%)
Women	1167 (70.3%)
Age, Median (Range) (data available, 1617, 97.4%)	54.0 (18–86)
*BMI, kg m* ^− 2^ * (data available, 1604, 96.6%)*	24.45 ± 4.66
< 18	40 (2.4%)
18–24.9	942 (56.7%)
25–29.9	448 (27.0%)
≥ 30	174 (10.5%)
*CCI (data available 1624, 97.8%)*	
CCI O	1418 (87.4%)
CCI 1	134 (8.1%)
CCI 2	56 (3.4%)
≥ CCI 3	14 (0.8%)
*Diagnosis code (data available, 1658, 99.9%)*	
C18 (colon cancer)	58 (3.5%)
C25 (pancreas cancer)	58 (3.5%)
C34 (bronchial cancer)	90 (5.4%)
C50 (breast cancer)	668 (40.2%)
C56 (ovarian cancer)	52 (3.1%)
C61 (prostate cancer)	94 (5.7%)
C71 (brain cancer)	36 (2.1%)
C81 (Hodgkin lymphoma)	46 (2.8%)
C85 (NHL)	63 (3.8%)
Other cancer types	493 (29.7%)
*Metastasis prevalence (data available, 1562, 94.1%)*	
Yes	333 (20.1%)
No	1185 (71.4%)
Clinically unclear	46 (2.8%)
*Baseline treatment (data available, 1609, 96.9%)*	
Active surveillance	10 (0.6%)
Active treatment	1071 (64.5%)
Follow-up < 1 y after EOT	389 (23.4%)
Follow-up > 1 y after EOT	104 (6.3%)
Follow-up > 5 y after EOT	35 (2.1%)
Treatment status unclear	51 (3.1%)

*BMI* body mass index, *CCI* Charlson Comorbidity Index, *NHL* non-Hodgkin lymphoma, *EOT* end of treatment. Data are presented as the number of patients and percentage of the total study population; data are included for diagnoses that reached the 2% threshold of the study population

### OTT Attendance and Adherence Data

**Fig. 2:**
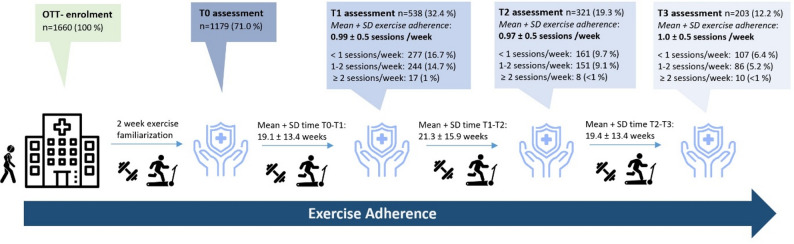
Data exercise adherence. *SD* standard deviation. T0 assessment—first assessment (including strengths and endurance tests and PROs) after 2 weeks of exercise familiarization. T1/T2/T3 assessments—repetition of T0 assessment which was planned 15 weeks after the initial or previous assessment

**Fig. 3:**
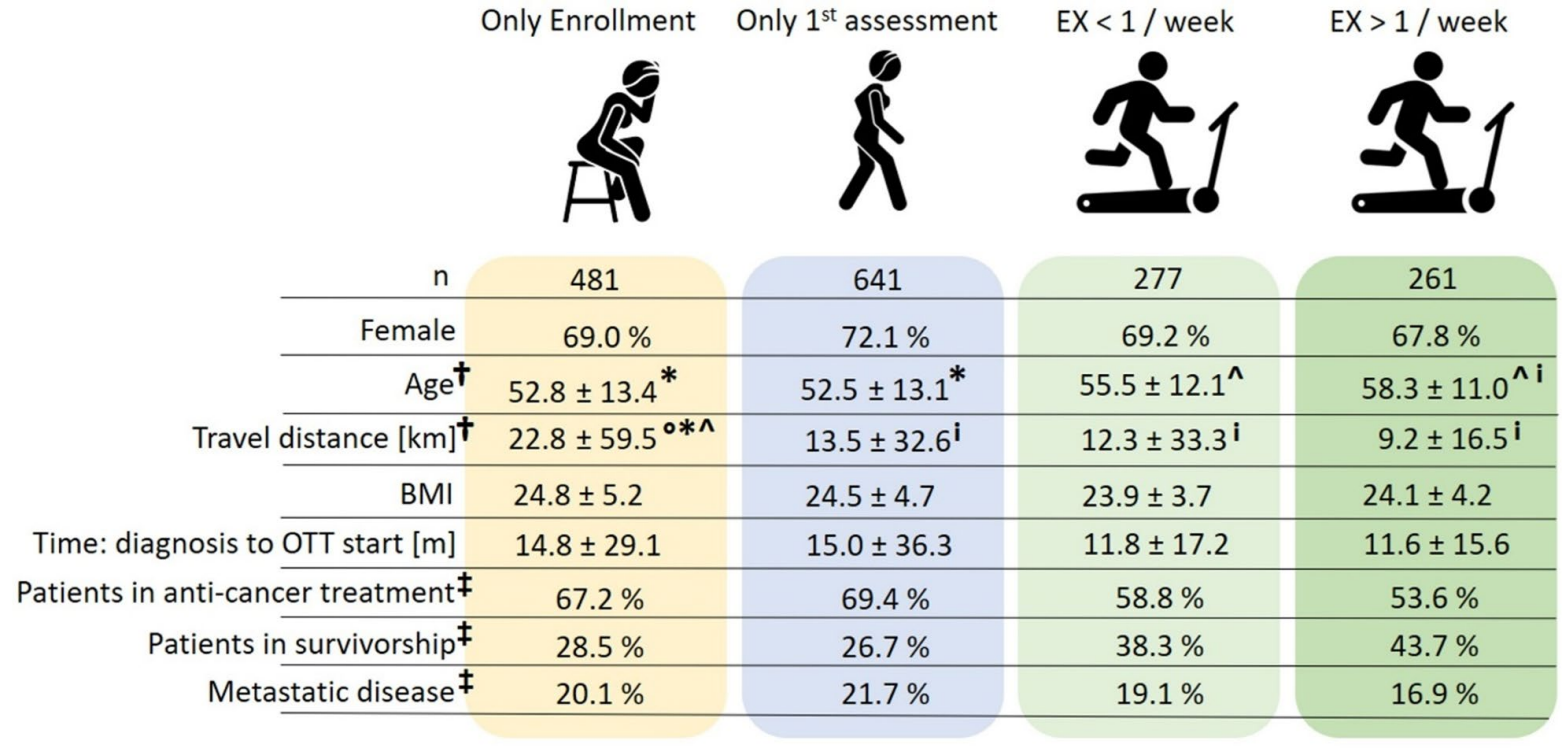
Demographic and disease-related differences across attendance subgroups. ^†^significant difference according to ANOVA (*p* < 0.05); ^‡^significant relation of variable and group (*p* < 0.05) [Chi²-Test]. °significant difference from EX < 1/week (*p* < 0.05) [Games–Howell post hoc test]; *significant differences in EX ≥ 1/week (*p* < 0.05); ^significant difference from only enrollment (*p* < 0.05); ^i^significant differences from only the 1st assessment (*p* < 0.05)

Following enrollment (*n* = 1660, 100%), a total of 1179 (68.6%) patients completed the T0 assessment after two weeks of exercise familiarization. Following a full exercise cycle, 538 (32.2%) patients reached the T1 assessment. The mean time between T0 and T1 was 19.1 ± 13.4 weeks (range: 2–95 weeks), with a mean frequency of 0.99 ± 0.5 exercise sessions/week (range: 0–3,4) (Fig. 2). Furthermore, *n* = 321 (19.3%) and *n* = 203 (12.2%) patients reached the T2 and T3 assessments, respectively. The mean number of exercise sessions was 0.97 ± 0.5 for T1-T2 and 1 ± 0.5 for T2-T3 (Fig. 2). A subgroup analysis revealed that older age (52 y vs. 58 y) and shorter travel distance to the exercise facility (22 km vs. 5 km) were associated with significantly greater exercise attendance (Fig. 3). In fact, weekly exercise attendance and patient age were positively correlated (*p* = 0.003). In addition, we observed different distributions of patients who were receiving anticancer treatment or were in cancer survivorship and patients with or without metastasis in the subgroup comparisons (Fig. 3).

### Exercise Efficacy Data and PROs

**Fig. 4:**
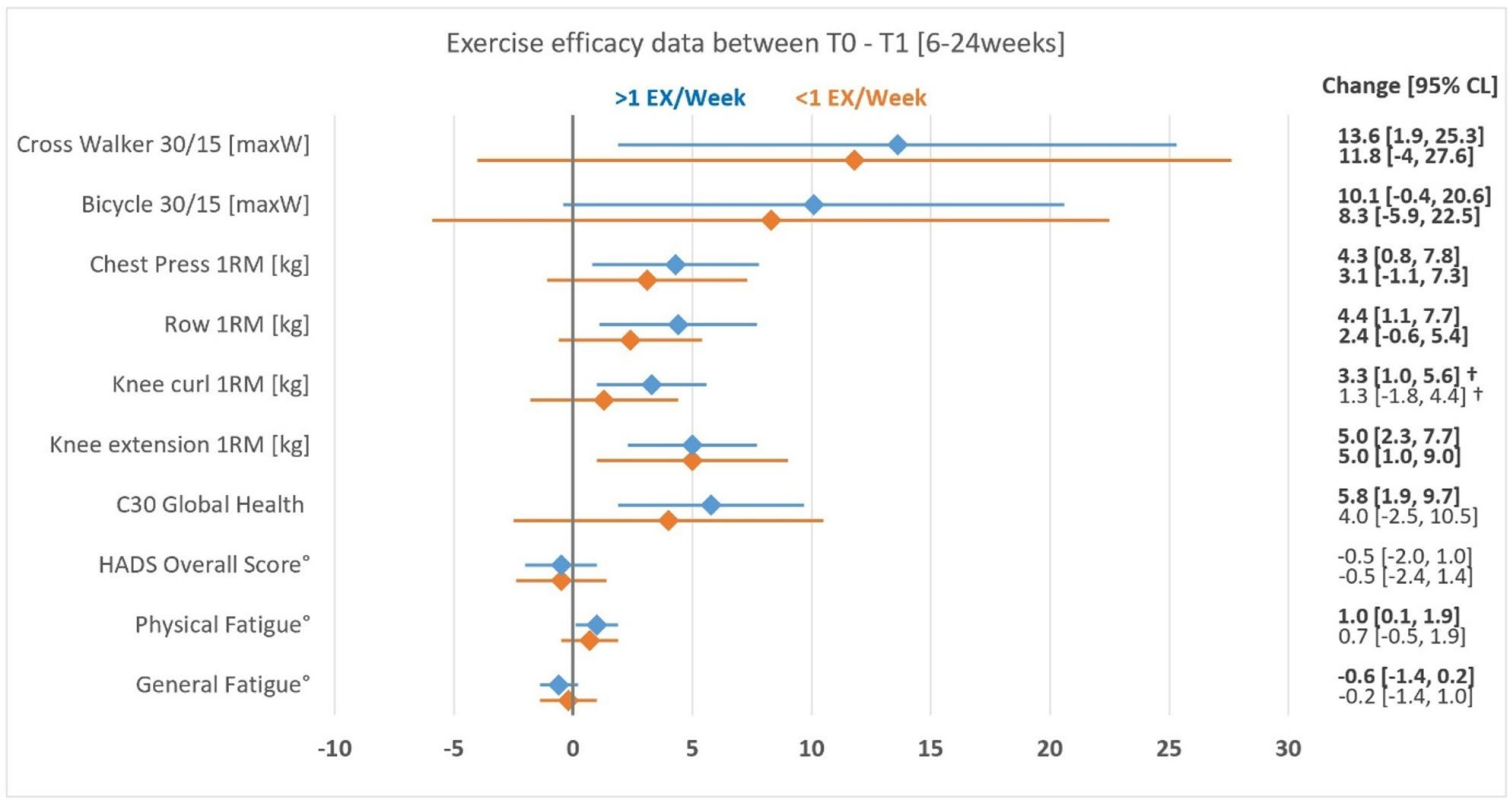
Data on exercise efficacy. °Reversed scales, indicating higher symptom burden with higher scores. ^†^Significant between-group intervention effect; 1RM = one repetition maximum; CL = confidence level; EX = exercise, HADS = Hospital Anxiety and Depression Scale

Exercising patients demonstrated improved endurance and strength performance in knee extension, flexion, chest press and row machines (*p* < 0.05 each; Fig. 4; Table 2). Additionally, exercising patients had improved quality of life but increased physical fatigue (*p* < 0.05 each; Fig. 4; Table 2). In the attendance subgroup analysis, knee flexion improved only in patients with an average of ≥ 1 exercise session per week and revealed a significant group difference from patients with an average of < 1 weekly exercise session (*p* = 0.02, Fig. 4). Both general fatigue, as assessed by the MFI-20 questionnaire, and global health status, as measured by the EORT-QLQ-C30 questionnaire, improved only in patients with an average of ≥ 1 exercise session a week (*p* < 0.01, Fig. 4). In contrast, physical fatigue, measured by the MFI-20 questionnaire, worsened as a symptom burden in patients with an average of ≥ 1 exercise session a week, whereas psychological distress scores did not change (Fig. 4; Table 2). Baseline differences among the attendance subgroups were observed in the domains of general fatigue (*p* = 0.05) and strength of knee extension (*p* = 0.026, Table 2).

**Table 2:** Exercise efficacy data: changes from the T0 to T1 assessments

	N	*Baseline*		*Post test*		*Mean change*		*Group diff* *Mean change*	
		Mean	SD	Mean	SD	Mean	95% CI	*P*	*P*
*General fatigue*									0.44
< 1 × EX/Week	68	12.4*	3.7	12.2	3.5	− 0.2	− 1.4 to 1.0	0.635	
≥ 1 × EX/Week	125	11.4*	3.3	10.8	3.4	− 0.6	− 1.4 to 0.2	**0.049**	
All EX patients	193	11.6	3.5	11.3	3.5	− 0.4	− 0.8 to 0.3	0.063	
*Physical fatigue*									0.53
< 1 × EX/Week	66	12.3	3.6	13	3.5	0.7	− 0.5 to 1.9	0.12	
≥ 1 × EX/Week	126	12.9	3.7	13.9	3.4	1	0.1 to 1.9	** < 0.01**	
All EX patients	192	12.7	3.6	13.6	3.5	0.9	0.4 to 1.5	** < 0.01**	
*HADS general*									1
< 1 × EX/Week	89	12.3	6.6	11.8	6.4	− 0.5	− 2.4 to 1.4	0.39	
≥ 1 × EX/Week	145	11	6.9	10.5	6.5	− 0.5	− 2.0 to 1.0	0.14	
All EX patients	234	11.5	6.8	11	6.5	− 0.5	− 1.0 to 0.1	0.10	
*C30 Global QoL*									0.49
< 1 × EX/Week	77	58.5	20.6	62.5	20.1	4	− 1.7 to 8.3	0.11	
≥ 1 × EX/Week	148	60.8	16.7	66.6	17.5	5.8	− 1.6 to 6.0	** < 0.01**	
All EX patients	225	60	18	65.2	18.5	5.2	2.2 to 7.1	** < 0.01**	
*1RM knee extension [kg]*									0.54
< 1 × EX/Week	58	21.5*	9.8	26.5	12	5	1.0 to 9.0	** < 0.01**	
≥ 1 × EX/Week	112	25*	9.5	30	11	5	2.3 to 7.7	** < 0.01**	
All EX patients	170	23.8	9.7	28.8	11.4	5	4.0 to 6.2	** < 0.01**	
*1RM knee* *flexion [kg]*									**0.02**
< 1 × EX/Week	60	24.1	9.3	25.4	7.9	1.3	− 1.8 to 4.4	0.79	
≥ 1 × EX/Week	126	25.5	8.6	28.8	10.1	3.3	1.0 to 5.6	** < 0.01**	
All EX patients	186	25	8.9	27.7	9.5	2.6	1.8 to 3.4	** < 0.01**	
*1RM Chest Press [kg]*									0.3
< 1 × EX/Week	57	23.2	11.3	26.3	11.2	3.1	− 1.1 to 7.3	** < 0.01**	
≥ 1 × EX/Week	105	26.8	12.6	31.1	13.1	4.3	0.8 to 7.8	** < 0.01**	
All EX patients	162	25.5	12.2	29.4	12.6	3.9	2.8 to 5.0	** < 0.01**	
*1RM Row [kg]*									0.13
< 1 × EX/Week	53	27.3	10.8	29.7	11.2	2.4	− 0.6 to 5.4	**0.01**	
≥ 1 × EX/Week	105	29.8	11.5	34.2	12.5	4.4	1.1 to 5.6	** < 0.01**	
All EX patients	158	29	11.2	32.7	12.2	3.7	2.5 to 4.9	** < 0.01**	
*30/15 Bicycle [maxW]*									0.64
< 1 × EX/Week	78	101	43.6	109.3	46.1	8.3	− 5.9 to 22.5	**0.12**	
≥ 1 × EX/Week	150	100.9	45.7	111	46.4	10.1	− 0.4 to 20.6	** < 0.01**	
All EX patients	228	100	44.5	109.3	45.9	9.6	5.9 to 13.2	** < 0.01**	
*30/15 crosser walker [maxW]*									0.65
< 1 × EX/Week	72	81.3	45.6	93.1	50.1	11.8	− 4 to 27.6	**0.03**	
≥ 1 × EX/Week	133	75.9	47.2	89.5	50.0	13.6	1.9 to 25.3	** < 0.01**	
All EX patients	205	77.8	46.9	90.7	50	12.8	9.3 to 16.4	** < 0.01**	

1RM = one repetition maximum; maxW = maximum watts; CI = confidence level; EX = exercise; HADS = Hospital Anxiety and Depression Scale. *Significant baseline difference (*p* < 0.05)

## Discussion

To our knowledge, this is the first published analysis reporting the successful integration and evaluation of an innovative, supervised exercise concept into the standard of clinical care for cancer patients. We observed that especially younger female cancer patients receiving anticancer treatment participated in the exercise program. Furthermore, we observed delayed engagement in exercise after diagnosis and that only one-third of the patients performed sustained exercise over a four-month period. The mean exercise attendance was only one session per week, highlighting a gap between real-world practice and recommendations of well-established exercise guidelines for cancer patients [2]. Crucially, we were able to identify patient age and travel distance as factors relevant to exercise attendance. Also importantly, patients who exercised demonstrated improved physical performance, which was more pronounced in patients with higher attendance rates. Similarly, key PRO measures, such as global health status or general fatigue, improved during OTT but only in patients with one or more than one weekly exercise session.

Our program primarily enrolled female patients. Most patients were diagnosed with breast, prostate or lung cancer and were receiving anticancer treatment (Table 1). Overall, the observed distribution of cancer types was relatively congruent with the yearly German cancer incidence rates [12]. Previous exercise studies reported comparable proportions of breast cancer patients ranging from 25 to 40%, suggesting a high degree of exercise interest in this particular cancer population [13]. Notably, colorectal cancer (CRC) patients (*n* = 58, 3.5%) seemed underrepresented in the OTT cohort reported herein, compared with 120,000 new cases/year reported in Germany [12]. CRC patients may suffer more complex postsurgical complaints that impede exercise initiation [13] or have a lower exercise referral rate through medical staff [14], ultimately resulting in lower participation rates in exercise trials [15]. Overall, this distortion of the general cancer population reduced the external validity of our results and limits the extents of the observation to herein reported, specific cancer populations.

Interestingly, we observed delayed exercise participation, with more than 40% of the patients enrolling in the OTT more than 6 months after their cancer diagnosis. This could be an indicator of increasing interest in exercise toward the completion of anticancer treatment [16] or the delayed onset of cancer-related impairments. Additionally, it is likely that newly diagnosed patients treated in and near Cologne were not immediately referred for OTT due to "a lack of awareness of the exercise program. We continuously promoted the OTT program with flyers, referral pathways as well as a web presence and encouraged medical staff for an early patient integration after cancer diagnosis. Within eight years, we observed a progressive increase in yearly OTT enrollments from 2012 to 2020 (*n* = 134 to *n* = 302), suggesting improved recognition of the OTT. From an oncological exercise therapy perspective, patients should be included in exercise programs as early as possible, i.e., ideally prior to anticancer treatment [2]. Through early exercise initiation, physical and psychosocial conditions may be maintained or even improved, and cancer-related impairments may be prevented or mitigated. As demonstrated by our study, many patients reported cancer-related impairments at enrollment, such as fatigue (69.7%), cancer-related cognitive impairments (CRCI, 41.1%), psychosocial distress (40.0%), chronic pain (~ 31.0%) and body weight loss (~ 21%) (Fig. S1). As a consequence of these observations, we established the OTT concept in the patients’ multidisciplinary treatment plan within the University Hospital Cologne and argued that this may lead to a reduction in hospitalizations and interruptions of anticancer treatment in the future [17–18].

Interestingly, exercise over a prolonged time period, with a mean of 19.1 ± 13.4 weeks, was performed by only 32% of the initially enrolled patients. In fact, 29% (*n* = 481) of the patients discontinued the exercise intervention within the first two weeks of familiarization. Presumably, some patients may have sought exercise counseling without actual exercise intent or may have continued exercising elsewhere [19]. These patients had a mean travel distance of 22.8 ± 59.5 km, which was significantly greater than that of all other attendance subgroups and may support this assumption (Fig. 3). In fact, travel distance has been reported to be a crucial factor in determining attendance at exercise programs in cancer patients [20]. Similarly, our data indicated that a lower travel distance was associated with greater exercise attendance (Fig. 3). In addition, we observed a greater distribution of patients with ongoing anticancer treatment and metastasis in both attendance subgroups, who discontinued exercise within the first two weeks or reached only one assessment (Fig. 3). These observations suggest greater barriers to engage or maintain exercise in patients with ongoing anticancer treatment or metastasis, as reported by De Lazzari et al. [21].

Nonetheless, 538 patients exercised for a mean duration of 19 weeks. On average, these patients exercised once a week according to the OTT, whereas only 3% of exercising patients (*n* = 17 out of 538) reached ≥ 2 OTT sessions per week (Fig. 2). This accounts for 1% of all enrolled patients (*n* = 17 out of 1660). This is in striking contrast to the well-established exercise guidelines and our recommendations of at least two supervised OTT exercise sessions per week [2]. The majority of exercising patients at OTT (*n* = 277, 51.5) had, on average, even less than one OTT session per week, highlighting challenges when the current exercise recommendations [2] were integrated into a real-world setting. The trend toward approximately one exercise/week did not change if patients exercised for 40 or 60 weeks (Fig. 2). Despite not reaching the recommended exercise prescription, the exercise program led to improvements in aerobic and strength performance and patients’ quality of life. Nonetheless, the mismatch of well-established exercise guidelines for cancer patients and the exercise attendance habits of patients with easily accessible, free-of-charge and tightly supervised OTT concepts illustrates the remaining challenges in delivering exercise therapy adequately within the standard of care. This challenge may be addressed by the inclusion of local, community-based exercise facilities or exercise programs with mixed supervised and home-based exercise. Unfortunately, our data structure did not allow an analysis of physical activity or exercise patterns outside the OTT framework. Interestingly, significantly higher baseline values of general fatigue were observed in the group of patients who had, on average, even less than one session per week (Table 2). In fact, fatigue symptoms are often described as potent barriers to exercise participation [22].

The relevance of increased exercise attendance was highlighted by the efficacy data as well. While strength and endurance performance improved significantly in all patients, the improvements in leg flexion strength revealed significant differences, favoring patients with greater exercise attendance. Patients with an average of ≥  1 weekly exercise session additionally showed improved general quality of life (EORTC-QLQ-C30) and reduced general fatigue scores (MFI-20), whereas patients with lower attendance rates showed no significant changes. These meaningful results concerning exercise efficacy highlight the importance of exercise interventions to maintain or improve physical performance and critical domains of PROs in cancer patients during anticancer treatment. Furthermore, these results illustrate the important relationship between exercise frequency and beneficial effects on physical performance and PROs and, thus, the importance of current exercise recommendations [2]. Notably, patients with higher attendance rates experienced greater physical fatigue. In this context, muscle soreness following especially intense exercise may play a crucial role. Most patients were inexperienced with structured exercise programs, eventually leading to muscular and cardiorespiratory adaptations increasing the perceived physical fatigue temporarily. Interestingly, the general fatigue burden on the other hand significantly decreased simultaneously in these patients.

### Clinical Implications

Consequently, future approaches that integrate exercise into the standard of care for cancer patients should target this potential mismatch of exercise attendance with concepts such as individualized exercise barrier analysis and counseling, motivational or behavioral interventions or innovative exercise approaches to maximize the beneficial effects of exercise. Methods such as mixed exercise programs with supervised and home-based or online exercise classes may lead to more appropriate convergence of patients’ exercise habits and guideline recommendations. A greater availability of oncology-specific exercise facilities close to home would be beneficial, but the financial aspect currently precludes such actions on a larger scale. The increasing integration of community-based exercise facilities seems to be a successful implementation method for cancer survivors [5]. In cancer patients receiving active anticancer treatment, however, exercise therapy should be performed with greater caution and delivered by trained health care professionals due to their vulnerable medical condition.

### Limitations

Our study has several potential limitations. First, this study did not include an exercise free control arm, limiting the inference of the OTT effects. Large, adequately powered RCTs are warranted to ensure the effects of the OTT. In addition, the high proportion of female breast cancer patients and underrepresentation of other common cancer types may limit the generalizability of the results. In addition, the referral-driven recruitment to the OTT and the self-chosen participation will most likely result in a selection bias, influencing the generalizability of the results. Moreover, some assessment tools were implemented in OTT after 2014, leading to smaller sample sizes for some outcomes.

## Conclusion

This report summarizes a multimodal analysis of eight years of experience after establishing an innovative, personalized exercise concept for cancer patients. On the one hand, we were able to demonstrate the beneficial effects of a structured exercise intervention on both physical measures and PROs. On the other hand, we observed an important mismatch between current guideline recommendations and the actual participation of cancer patients in the OTT, a substantial delay between diagnosis and exercise initiation and relatively short exercise attendance. Future exercise research should address these discrepancies and build on innovative approaches to enhance participation, earlier timing of exercise engagement in the cancer trajectory and increased exercise attendance rates to deliver exercise therapy most effectively. Future research should additionally examine how to approach currently underrepresented groups such as CRC patients effectively and translate this knowledge into effective routine care structures to utilize exercise interventions adequately for all cancer patients.

## Supplementary Information

Below is the link to the electronic supplementary material.

Supplementary Material 1

## Data Availability

Data will be made available on reasonable request. Proposals for data should be directed to the corresponding author (timo.niels@uk-koeln.de).

## Abbreviations

1RM: One repetition maximum
ACSM: American College of Sport Medicine
BMI: Body mass index
CCI: Charlson Comorbidity Index
CIPN: Chemotherapy-induced peripheral neuropathy
CL: Confidence level
CRCI: Cancer-related cognitive impairments
EOT: End of treatment
EX: Exercise
HADS: Hospital Anxiety and Depression Scale
IQRs: Interquartile ranges
maxW: Maximum Watt
MFI: Multidimensional Fatigue Inventory
NHL: Non-Hodgkin Lymphoma
OTT: Oncological exercise therapy
PROs: Patient-reported outcomes
SD: Standard deviation
VAS: Visual analog scale

## Declarations

### Patient Involvement

Patients were involved in the design and/or conduct of this research.
