# Correction: Innovative Exercise in Routine Cancer Care: Insights from Eight Years of Integrated Oncological Exercise Therapy (OTT)

**DOI:** 10.1186/s40798-026-01046-5

**Published:** 2026-06-15

**Authors:** Timo Sonntag, Ariana Safi, Vera Coutellier, Anna Lorenz, Philipp Zimmer, Eva M. Zopf, Fiona Streckmann, Lars Gerland, Petra Wirtz-Derksen, Anja Großek, Anne Kollikowski, Constanze Handmann, Stefanie Siebert, Paul J. Bröckelmann, Christian P. Pallasch, Wilhelm Bloch, Thomas Elter, Michael Hallek, Damir Zubac, Freerk T. Baumann

**Affiliations:** 1 Faculty of Medicine and University Hospital Cologne, Department I of Internal Medicine, Center for Integrated Oncology Aachen Bonn Cologne Duesseldorf, University of Cologne, Cologne, Germany; 2 Division of Performance and Health, Institute for Sport and Sport Science, Technical University Dortmund, Dortmund, Germany; 3 Mary MacKillop Institute for Health Research, Australian Catholic University, Melbourne, VIC Australia; 4 Department of Medical Oncology, Cabrini Cancer Institute, Cabrini Health, Melbourne, VIC Australia; 5 Department of Sport, Exercise and Health, University of Basel, Basel, Switzerland; 6 Oncology, University Hospital Basel, Basel, Switzerland; 7 Department of Molecular and Cellular Sports Medicine, Institute of Cardiovascular Research and Sports Medicine, German Sport University Cologne, Cologne, Germany; 8 Comprehensive Cancer Center Mainfranken, University Hospital of Würzburg, Würzburg, Germany

**Correction: Sports Medicine - Open (2026) 12:22** 10.1186/s40798-026-00988-0

The original article [1] displays an obsolete version of Figure 1; the updated version (Fig. 1) is shown ahead in this Correction article.

**Fig. 1:**
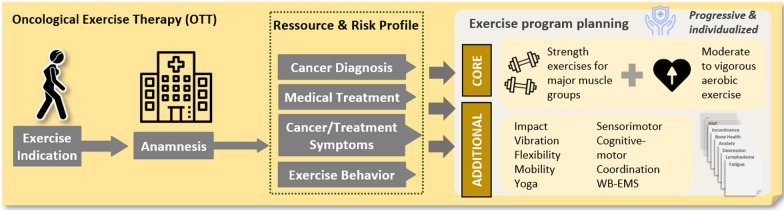
Oncological Exercise Therapy (OTT) concept
