# Supplementary material for: Innovative Exercise in Routine Cancer Care: Insights from Eight Years of Integrated Oncological Exercise Therapy (OTT)
Source: Sports Med Open. 2026 Mar 4;12:22. doi: 10.1186/s40798-026-00988-0 (PMC12961029; doi:10.1186/s40798-026-00988-0)
Supplement: Supplementary file 1 — Supplementary Material 1 [file 40798_2026_988_MOESM1_ESM.docx]

*Original Article*

**Innovative exercise in routine cancer care: Insights from eight years of integrated Oncological Exercise Therapy (OTT).**

Running head: Exercise as routine care: Oncological exercise therapy (OTT)

T Sonntag^1^, A Safi^1^, V Coutellier^1^, A Lorenz^1^, P Zimmer^2^, EM Zopf^3,4^, F Streckmann^5,6^, L Gerland^1^, P Wirtz-Derksen^1^, A Großek^7^, A Kollikowski^8^, C Handmann^1^, S Siebert^1^, PJ Bröckelmann^1^, CP Pallasch^1^, W Bloch^7^, T Elter^1^, M Hallek^1^, D Zubac^1^, FT Baumann^1^

^1^ University of Cologne, Faculty of Medicine and University Hospital Cologne, Department I of Internal Medicine, Center for Integrated Oncology Aachen Bonn Cologne Duesseldorf

^2^ Division of Performance and Health, Institute for Sport and Sport Science, Technical University Dortmund, Dortmund, Germany

^3^ Mary MacKillop Institute for Health Research, Australian Catholic University, Melbourne, Victoria, Australia

^4^ Cabrini Cancer Institute, Department of Medical Oncology, Cabrini Health, Melbourne, Victoria, Australia.

^5^ Department of Sport, Exercise and Health, University of Basel, Basel, Switzerland.

^6^ Oncology, University Hospital Basel, Basel, Switzerland.

^7^ Department of Molecular and Cellular Sports Medicine, Institute of Cardiovascular Research and Sports Medicine, German Sport University Cologne, Germany

^8^ Comprehensive Cancer Center Mainfranken, University Hospital of Würzburg, Würzburg, Germany

*Corresponding Author:*

Timo Sonntag, PhD

Center of Integrated Oncology, Oncological Exercise Medicine,

Kerpener Str. 62, 50937 Cologne, Germany. Tel: +49 22147842646,

Fax: +49 22147897191, Email: timo.niels@uk-koeln.de

**Supplementary Material (Tables/Figures)**

**Supplementary Figure 1*.*** Data on reported disease/treatment related impairments at time of anamnesis. Abbreviations: CRCI - cancer related cognitive impairment; CIPN – chemotherapy induced Polyneuropathy; BW – Body weight; Data available for n=1598 (96.0 %) of the full sample, data missing in n=62 (3.7 %). ***** Fatigue and Psychosocial Distress were measured in n=1000 (60.1 %) and n=1050 (63.2 %) patients and displayed in % from the respective sample size.

**Supplementary Table 1**. Anti-cancer treatment

| *N* (1660, total study population) | No. |  |
| --- | --- | --- |
| Baseline treatment (*data available, 1609, 96.9* %) |  |  |
| Chemotherapy at baseline |  |  |
| *Completed treatment* | 464 (28.0 %) |  |
| *Under treatment* | 729 (43.9 %) |  |
| Immunotherapy at baseline |  |  |
| *Completed treatment* | 101 (6.1 %) |  |
| *Under treatment* | 266 (16.0 %) |  |
| Radiotherapy |  |  |
| *Completed treatment* | 460 (27.7 %) |  |
| *Under treatment* | 181 (10.9 %) |  |
| Surgery at baseline |  |  |
| *Completed treatment* | 1045 (63.0 %) |  |
| *Under treatment* | 20 (1.2 %) |  |
| AHT/ADT at baseline |  |  |
| *Completed treatment* | 44 (2.7 %) |  |
| *Under treatment* | 244 (14.7 %) |  |
| SCT at baseline |  |  |
| *Completed treatment* | 34 (2.0 %) |  |
| *Under treatment* | 1 (0.1 %) |  |

**Abbreviations**: AHT - antihormone therapy; ADT – Androgen deprivation therapy; SCT - stem cell transplantation. Data are presented no. of participants and % of total study population;

***Supplementary Table 2.*** *Data on patients exercise access pathways.*

| *N* (1660, total study population) | No. |  |
| --- | --- | --- |
| *Time between diagnosis to OTT enrollment* (*data available,* 1474, 88.8 %, *Median (Range) in months* | 6 (0-492) |  |
| *0 – 3 months* | 438 (26.4 %) |  |
| *4 – 6 months* | 333 (20.0 %) |  |
| *7 – 12 months* | 332 (20.0 %) |  |
| *> 12 months* | 371 (22.3 %) |  |
| *Unclear* | 186 (11.2 %) |  |
| *Patients during active treatment, Median (Range), months* | 4 (0-492) |  |
| *Patients with metastasis, Median (Range), months* | 7 (0-492) |  |
| *Cancer survivors, Median (Range), months* | 11 (0-379) |  |
| Distance from home to OTT (data available, 1575, 94.9 %), Median (Range) in km | 6,9 (0-494) |  |
| *< 5km* | 541 (32.6 %) |  |
| *5-9,9km* | 459 (27.7 %) |  |
| *10-14,9 km* | 248 (14.9 %) |  |
| ≥ *15 km* | 322 (20.4 %) |  |
| Pathways of OTT-referral (data available, 792, 47.7 %) |  |  |
| *Physician recommendation* | 185 (11.1 %) |  |
| *In-hospital promotion (University Hospital Cologne)* | 212 (12.8 %) |  |
| *Other clinics near Cologne* | 152 (9.1 %) |  |
| *Recommendation from social environment* | 83 (5.0 %) |  |
| *Internet/TV* | 78 (4.7 %) |  |
| *Other* | 82 (4.9 %) |  |

**Abbreviations:** OTT - Oncological exercise therapy; Data are presented no. of participants and % of total study population

**Supplementary Methods**

**Comprehensive exercise and assessment description**

***Exercise description***

Based on a comprehensive medical history and initial examination (see Measurement Methods), all patients received an individualized exercise program. The duration and content of the program were tailored to the cancer type, medical treatment, disease- and treatment-related side effects, prior experience, and the personal goals and interests of the patient. A shared decision-making process between patients and therapist was used to design the concrete exercise program. If no contraindications (see Contraindications) existed and if possible, all patients underwent basic training. Basic training consisted of progressive strength and endurance exercises. The facility was equipped with a chipcard-driven Milon training circle (Milon GmbH), containing following exercises devices: (1) Chest press, (2) Row (3) Back extension (4) Abdominal crunch (5) Leg curl (6) Leg extension (7) three Crosstrainer and (8) three Bicycle-ergometer. Additionally, an area with mats, elastic bands, free weights, a vibration plate (Galileo GmbH) and material for balance training were available

The exercise weight for the strength training devices was determined based on a hypothetical one-repetition maximum test (h1RM) ^(1,2)^ the planned training cycle, and the RPE scale (target range 6). The training cycles for strength training are visible in Table 1.

Table 1: Strength training cycles

| **Training cycles** | **Intensity**  **(% of the h1RM)** | **RPE Scale**  **(1-10)** | **Repetitions** | **Sets** | **Sessions/**  **Week** | **Sessions/**  **cycle** |
| --- | --- | --- | --- | --- | --- | --- |
| **Familiarisation** | - | 5 | 20 | 2 | 2 | 2-4 |
| **Cycle I: Strength endurance** | 50-60 | 6 | 20 | 2 | 2 | 30 |
| **Cycle II: Hypertrophy Training** | 70-80 | 7 | 8-12 | 2 | 2 | 30 |
| **Cycle III:**  **Excentric Overload** | 70-80 + 30 (ex) | 8 | 8-12 | 2 | 2 | 30 |

The endurance exercises were controlled with the peak work capacity (max. Watts), retrieved a cardiopulmonary exercise testing (CPET) following the 30/15 protocol specifically designed for cancer patients. Patients start the CPET with 30 Watts and the load continuously increases by 15 Watts per minute until patient’s exhaustion. The training cycles for endurance exercises are visible in Table 2.

| **Training cycles** | **Intensity**  **(% of maxW)** | **Borg-Scale**  **(6-20)** | **Duration** | **Sessions/**  **Week** | **Sessions/**  **cycle** |
| --- | --- | --- | --- | --- | --- |
| **Familiarisation** | - | 11-5 | 10-20min | 2 | 2-4 |
| **Cycle I: Moderat** | 50-60 | 13-15 | 16-20min | 2 | 30 |
| **Cycle II: Intense** | 70-80 | 16-17 | 16-20min | 2 | 30 |

Table 2: Endurance exercises cycles

The exercise cycles are not designed as bottom-up cycles. Depending on the patients goal setting and medical situation, the exercise cycles were prescribed. For instance, a pancreatic cancer patients facing body and muscle mass loss, may exercise in cycle 2-3 within the strength exercises and cycle 1 in the endurance exercises.

The whole exercises were prescribed as progressive exercise training. Patients should report, if the given weight or loads were not matching the aimed value of the Borg-scale, indicating necessary adaptions in weights or watt loads.

If specific deficits are present, additional training modules are determined. In each individual case, it is decided whether basic training, a combination of basic training and module training, or only module training will be carried out. The supplementary training modules are presented in Table 2.

Table 2: Exercise modules

| **Exercise module** | **Core training possible (adaptations necassary) ?** | **Supplementary exercises?** |
| --- | --- | --- |
| **Urinary incontinence** | **Yes**  (if necessary, omit devices in case of incomplete scar healing and severe urinary incontinence). | Sphincter training with special attention to the pelvic floor  (48 hours after catheter removal) |
| **Chemotherapy-induced polyneuropathy** | **Yes** | Vibration training  (provided there are no osteolyses, osteosyntheses, hip TEPs, fractures of the lower extremities, thromboses, ulcers on the foot)  and/or sensorimotor training |
| **Cachexia** | **Yes**  (especially cycle II and III, endurance training moderate intensity) | - |
| **Fatigue** | **Yes, adapted**  (depending on the expression, reduce intensity) | - |
| **Bone metastasis** | **Depending on the degree**  (if there is a risk of bone fracture, omit exercises of the corresponding body region) | Group training with gymnastic strengthening exercises, sensomotoric/coordination, body awareness  (only if basic training is not possible) |
| **Lymphedema** | **Yes, adapted**  (especially cycle I and II, depending on symptom development) | Nordic walking and water therapy recommended |
| **Osteoporosis** | **Depending on the severity**  (in osteopenia: cycle II and III,  in case of mild osteoporosis: cycle I) | Exercise program with isometric strengthening exercises, balance training and impacts.  (only for moderate and severe osteoporosis (with fractures). |
| **Side effects of the hormone-treatment** | **Yes**  (especially cycle II and III) | Additional endurance training recommended |

The duration of the intervention depends on the disease- and treatment-related side effects of the patients but typically ranges from 3 to 12 months. Intermediate assessments are conducted for all patients after every 30 training sessions (~15 weeks). For patients with existing polyneuropathy, intermediate assessments are conducted after 24 training sessions as part of the module training.

**Contraindications**

Note: The contraindications listed here are cancer-specific contraindications. Orthopedic, cardiac, and psychological contraindications must also be considered.

Absolute Contraindications

Note: Absolute contraindications do not mean a prohibition of movement or inactivity. Training at OTT is contraindicated, but daily activities are not excluded.

• Acute bleeding or a strong tendency to bleed

• Platelet count below 10,000/µl

• Bleeding tendency or signs between 10,000 and 20,000/µl platelets

• Severe pain and exacerbation of pain with movement

• Fever or a temperature above 38°C that is not considered B-symptoms

• Potentially communicable infections (to protect other patients)

• The first 48 hours after concurrent administration of Herceptin and chemotherapy (risk of arrhythmias)

• Hemoglobin levels below 8g/dl of blood in combination with dizziness

Relative Contraindications

Note: Relative contraindications must be considered by the therapist on an individual basis before the safety of training at OTT can be determined.

• On the days when and 24-48 hours after the administration of acutely heart- or kidney-damaging chemotherapy (no strenuous interventions)

• Nausea or vomiting

• Impairment of consciousness and confusion

• Hemoglobin levels below 8g/dl of blood

• Dizziness

• Irradiated structures located in the pressure point area of the training equipment

• Brain metastases (no strenuous interventions due to the risk of seizures)

• Liver metastases (no strenuous interventions due to portal vein blockages and the resulting risk of cardiovascular decompensation)

**Assessment description**

Assessments to evaluate muscle strength, aerobic performance, quality of life, anxiety, and depression were conduct for all patients after every 30 training sessions (~15 weeks).

**Measurement Methods**

Hypothetical One-Repetition Maximum

On the strength exercise machines, patients performed a One-repetition-maximum (1RM) or in most cases of medical limitations (s.a. previous surgery) a hypothetic-one-repetition-maximum (h1RM). The h1RM is a standardized dynamic strength test in which maximum strength is calculated based on the training weight and the completed repetitions. Patients were assessed with the maximum weight that they could perform eight repetitions with. Afterwards the used weight and the amount of repetitions were used in accordance with Gießing’s formula ^(5)^ to calculate the h1RM, which is reported be very close to the real 1RM ^(6).^

30/15 maxWatt endurance test

To assess aerobic performance, patients performed a cardiopulmonary exercise testing on a bycicle ergometer. They started with a load of 30 Watts and increasing by 15 Watts every minute until subjective perceived exertion (Borg scale >18). The test was performed with concomitant measurement of the heartfrequency and the blood pressure (every 2 minutes).

Questionnaires

To gather anthropometric, sociodemographic, and medical data, as well as disease-related events, a **self-constructed medical history** form was used.

Health-related quality of life is assessed using the internationally recognized and repeatedly validated "**Quality-of-Life Questionnaire Core-30" from the European Organisation for Research and Treatment of Cancer (EORTC-QLQ-C30)**.

To assess fatigue symptoms, the **"Multidimensional Fatigue Inventory" (MFI-20)** is used.

Anxiety and depression were assessed using the **"Hospital Anxiety and Depression Scale" (HADS).** The questionnaire has already been extensively validated. Patients with a noticeable score are informed about the possibility of psycho-oncological support within the University Hospital Cologne.

**References**

1. LeSuer DA, McCormick JH, Mayhew JL, Wasserstein RL, Arnold MD. The Accuracy of Prediction Equations for Estimating 1-RM Performance in the Bench Press, Squat, and Deadlift. J Strength Cond Res. 1997;11(4):211. doi:10.1519/1533-4287(1997)011<0211:TAOPEF>2.3.CO;2
2. Gießing J. Trainingsplanung und -steuerung beim Muskelaufbautraining: Das Konzept vom individuellen hypothetischen Maximalgewicht (h1RM) als methodische Alternative. 2003.
